# The Use and Potential of Biomedical Detection Dogs During a Disease Outbreak

**DOI:** 10.3389/fmed.2022.848090

**Published:** 2022-04-04

**Authors:** Michele N. Maughan, Eric M. Best, Jenna Dianne Gadberry, Caitlin E. Sharpes, Kelley L. Evans, Calvin C. Chue, Patrick Lawrence Nolan, Patricia E. Buckley

**Affiliations:** ^1^Excet, Inc., Springfield, VA, United States; ^2^Penn State Harrisburg, Harrisburg, PA, United States; ^3^Intrinsic24, LLC, Hayden, ID, United States; ^4^Biochemistry Branch, U.S. Army DEVCOM Chemical Biological Center, Aberdeen Proving Ground, MD, United States; ^5^Tactical Directional Canine Systems, LLC, Smithsburg, MD, United States

**Keywords:** biomedical detection dog (BMDD), canine, olfactory science, training aid delivery device (TADD), COVID-19, volatile organic compound (VOC), volatilome

## Abstract

Biomedical detection dogs offer incredible advantages during disease outbreaks that are presently unmatched by current technologies, however, dogs still face hurdles of implementation due to lack of inter-governmental cooperation and acceptance by the public health community. Here, we refine the definition of a biomedical detection dog, discuss the potential applications, capabilities, and limitations of biomedical detection dogs in disease outbreak scenarios, and the safety measures that must be considered before and during deployment. Finally, we provide recommendations on how to address and overcome the barriers to acceptance of biomedical detection dogs through a dedicated research and development investment in olfactory sciences.

## Introduction

Detection dogs have played a role in society since the Middle Ages, depicted wearing armor alongside knights and the familiar howl of the bloodhound as it tracks down criminals or missing people. In modern society, detection dogs are most often seen in a law enforcement capacity, screening people, luggage, vehicles, and cargo for contraband. However, a trend is emerging in which dogs’ olfactory abilities are being harnessed to not only detect a growing list of contraband, but also in an increasing number of fields and applications completely outside of law enforcement. A small selection of these detection disciplines is listed in Table 1.

**TABLE 1:** A selection of examples demonstrating the growing list of detection dog disciplines.

Contraband	
	○ Explosives
	○ Narcotics
	○ Firearms
	○ Currency
	○ Agricultural products
	○ Exotic animals or animal products
	○ Lithium-Ion Batteries
Live human	
	○ Search and rescue
	○ Patrol/Apprehension
	○ Tracking/Trailing
Forensics	
	○ Human remains/Cadaver (dead humans)
	○ Bodily fluids
	○ Arson/Accelerant/Fire Inspection
	○Human scent
Conservation	
	○ Endangered/Threatened species
	○ Site surveys to assess the effect of infrastructure on animal habitats
Electronics (storage devices, mobile phones)*	
Hobby/Sport	
Biomedical (Table 2)	

*Falls into both the forensics and contraband detection categories.

There are approximately 10,000 law enforcement working dogs in the United States amongst the military, federal, local, and state police agencies (1). These working dogs are present on our military bases, in our transportation hubs (e.g., train stations, airports, seaports), and on the streets of every major city in the United States. Another way of looking at these numbers and their geographical and situational distribution is to see the potential of having a network of highly adaptable sensors all throughout the country, able to detect any threat with a reproducible odor. The current COVID-19 pandemic has shown that globally, we were not prepared to handle an outbreak of that magnitude, especially of an unknown pathogen. Since there was no immediate understanding of the infectivity and transmissibility of the virus, there was a willingness to look outside the typical methods for pathogen detection and identification; potentially repurposing prophylactics, treatments and/or diagnostic/detection equipment. Ultimately, there was a need to investigate our most primitive (but not unsophisticated), yet reliable form of detection, canine olfaction.

Much of what is needed to address and terminate an outbreak is pathogen-dependent. Typically, the pathogen must be isolated, identified, cultured, its genetic material sequenced, and only then can the scientific community begin to develop effective vaccinations, therapeutics, and diagnostics. In the meantime, the community follows the “Swiss Cheese” model, relying on personal responsibilities such as personal protective equipment (PPE) (e.g., masks), social distancing, frequent handwashing, and cough etiquette (2) to combat the general spread of germs, but not the detection of the pathogen. But what can be effective while we wait for the scientific community to ramp up, is canine-based detection as canines only rely on the pathogen or the disease-state odor. We do not even need to necessarily have that odor’s volatile organic compound (VOC) profile characterized, we just need a way to safely capture/reproduce, store, and present the odor to the detection dogs. This odor detection scenario is obviously a gross oversimplification of the process, but it is currently the most straightforward of all of our detection capabilities. One should note that at this time Biomedical Detection Dog (BMDD) capabilities are considered detection or screening tool and not diagnostic technology. The distinction being that to be a diagnostic, BMDDs would need approval from the United States Food and Drug Administration (FDA) (3).

Beginning with the 1989 (4) and 2001 (5) case reports of patients’ pet dogs causing concern due to the excessive sniffing their dogs conducted at suspicious moles that were later determined to be cancerous, the ability of dogs to sniff out disease has grown from anecdotal to a full-fledged scientific discipline. Now BMDDs work as part of research teams in prestigious academic institutions such as the University of Pennsylvania’s PennVet Working Dog Center (established 2012), detecting ovarian cancer, sinonasal inverted papilloma, COVID-19, Spotted Lanternfly infestations, biofilms, and chronic wasting disease (6). An established body of literature exists demonstrating the effectiveness of dogs and their ability to detect the VOC signatures associated with disease including, but not limited to, toxigenic *Clostridium difficile* in stool (7), lung and breast cancers in breath (8), four different bacteria causing urinary tract infections in patient urine samples (9), bovine viral diarrheal virus (BVDV) infected cell-cultures (10), supernatant from *Pseudomonas aeruginosa* cultures (11), parasitic *Plasmodium falciparum* (malaria) infection using patient clothing (12), prostate cancer in urine (13), ovarian cancer in blood (14, 15), type 1 diabetes (16), and Parkinson’s disease (17) in sebum. Disease detection by canines has been systematically reviewed by Moser and McCulloch (18), Edwards et al. (19), Cambau and Poljak (20), and Salgirli Demirbaş et al. (21) and reported to be a scientifically sound method of detection.

BMDD history can be roughly categorized into three periods of time: the beginning starting with the 1989 case report of melanoma and culminating in 2010 with the Moser et al. review “Canine scent detection of human cancers: A review of methods and accuracy” wherein six published studies on canine detection of human cancers were reviewed in depth. This beginning period focused nearly exclusively on canine detection of cancer. The next period runs approximately from 2010 to 2020 in which the field of biomedical detection dogs expands beyond cancer and into the variety of subdisciplines (Table 2). This ten-year period is marked by an explosion of canine detection research resulting in a growing list of detectable human diseases by BMDDs and BMDDs able to detect **virus** [bovine viral diarrhea virus (10)], **bacteria** [C. difficile (7), Escherichia coli, Klebsiella pneumoniae, Enterococcus faecalis, and Staphylococcus aureus (9)], **pests** (brown tree snakes (22), palm weevils (23), gypsy moths (24), longhorn beetles (25), termites (26), bed bugs (27), and quagga and zebra mussels (28), fouling agents [catfight off-flavoring compounds (29), microbial growth in buildings (30)], **animals important to conservation efforts** [grizzly and black bears (31), brown bears (32), geckos and tuataras (33), tortoises (34), quolls (35), jackals (36), giant bullfrogs (37), wolves (38), rabbits (39), rock ptarmigans (40), bats (41), koalas (42), kit foxes (43), tigers (44), cougars (45), cheetahs (46), bobcats (47), and gorillas (48)], and **disease odor directly on humans** [Parkinson’s (49), epilepsy (50), diabetes (16, 51)].

**TABLE 2:** Subdisciplines within the biomedical detection dog field and examples of the diseases/pathogens/pests they detect.

Biomedical detection dogs*	
DISCIPLINE	EXAMPLES
Medical detection	Non-infectious:
(Detects disease state, i.e., signature volatilome or change in volatilome produced by infected hosts)	• Cancers: Melanoma (113)
	• Altered Metabolic Status: Diabetes (16)
	Infectious: Malaria (12)
Agricultural disease detection	Potato virus Y (PVY), the etiological agent of Potato Tuber Necrotic Ringspot Disease (PTNRD) (114)
Biological detection	Bovine Viral Diarrheal Virus (BVDV) (10)
(Detects pathogen)	
Pest/Invasive species detection	Pests: Bed bugs
	Invasive Species: Asian longhorn beetle, *Anoplophora glabripennis* (25)

*While detecting a biological organism, for the purposes of this review, biomedical detection dog (BMDD) specifically does not include conservation, forensic, and live human detection dogs as these detection disciplines would not be directly relevant to disease detection during an outbreak scenario.

The third period of BMDD history began in early 2020, coinciding with the SARS-CoV-2 global pandemic. Research groups from around the world, already deeply knowledgeable about the abilities of canines to detect human diseases, embarked on proof-of-concept studies to determine if BMDDs would be able to detect a human disease caused by a virus, in the midst of a pandemic caused by said virus. Based upon BMDD detection of the wide variety of human diseases and BMDD detection of a virus (BVDV), all of the evidence supported this as a valid next step for canine detection. The novel aspect of what was being attempted was BMDD detection of a human disease (COVID-19) caused by a virus (SARS-CoV-2). The global success of the COVID-19 detection dogs demonstrated the efficacy of BMDD detection of virus-induced human disease, but more significantly, it demonstrated the potential for BMDDs during a disease outbreak.

Five significant COVID-19 BMDD research highlights over the past 2 years are that these dogs:

(1) were trained, tested, and evaluated at research institutions or utilized in some capacity in at least twenty-five countries [Argentina (52), Austria (53), Australia (54), Belgium (55), Brazil (56), Cambodia (57), Canada (58), Columbia (59), Chile (60), Czech Republic (61), El Salvador (62), Finland (52), France (63), Germany (64–66), India (67), Iran (68), Italy (69), Lebanon (52), Russia (70), South Africa (71), Switzerland (72), Thailand (73), United Arab Emirates (74), United Kingdom (75), United States of America (76)] and, when assessed, demonstrated results in sensitivity and specificity, ranging from 65 to 100% and 76 to 99% (77), respectively, illustrating the consistency and robustness of their detection accuracy despite the differing training methodologies employed,
(2) were deployed in at least four countries (Finland, Lebanon, UAE, and United States) screening people for COVID-19 in airports (78, 79),
(3) demonstrated the ability in one study to achieve detection sensitivities greater than the gold standard real-time polymerase chain reaction (RT-PCR) and in less time (80), demonstrating their potential role in medical diagnostics,
(4) distinguished COVID positive from COVID negative samples with similar efficacy regardless of body fluid sampled (i.e., saliva, urine, and sweat) (66) demonstrating the range of non-invasive samples that BMDDs are capable of utilizing in a pandemic, and
(5) in one study, were able to differentiate SARS-CoV2 infections from infections with other novel coronaviruses, influenza viruses, parainfluenza viruses, an adenovirus, a rhinovirus, a metapneumovirus (HMPV), and respiratory syncytial virus (RSV)—all etiological agents common to respiratory tract infections (65), thus demonstrating the potential for BMDDs to aid in the triage and differential diagnosis process.

Utilizing the COVID-19 BMDDs as an example, one of the first questions to address during a disease outbreak would be if the BMDDs were able to detect the pathogen or disease and to what extent. The sensitivity and specificity of COVID-19 BMDDs has been reviewed in depth (77, 81–84) and the answer to this question is an overwhelming “yes.” Now that it has been irrefutably established that BMDD detection of a pandemic human disease caused by a virus is not only possible but that it is faster and more sensitive than our gold standard diagnostics, the question is what is the potential for BMDDs going forward for the next disease outbreak and what are some of the considerations that should be made around BMDD deployment.

### What Is a Biomedical Detection Dog?

For the purposes of this review, the term biomedical detection dog (BMDD) is an all-inclusive term to include: medical detection dogs that detect diseases in humans, agricultural disease detection dogs, and biological detection dogs that detect the microorganisms or etiological agents themselves, and to a smaller extent pest/invasive species detection dogs that detect primarily nuisance plant, animal, or insect life or invasive species as defined by Executive Order 13,112 “…as an alien species whose introduction does or is likely to cause economic or environmental harm or harm to human health” (Table 2).

**FIGURE 1:**
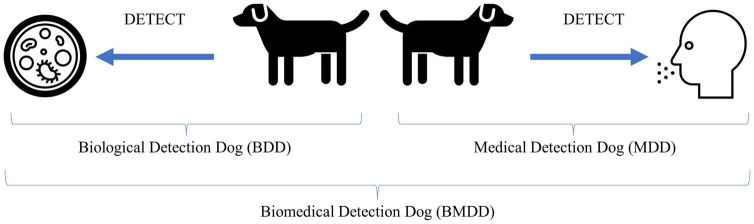
Illustration of biological and medical detection dogs and what they detect. The Biological Detection Dog (BDD) detects the odor of (or odors associated with) the pathogen or etiological agent, while the Medical Detection Dog (MDD) detects the odor of (or odors associated with) the disease state produced by an infected host in response to the pathogen or the altered volatilome due to disease not caused by an infectious agent. Together, BDDs and MDDs can be categorized as Biomedical Detection Dog (BMDDs).

One should note that these disciplines are not mutually exclusive and certain diseases and canine training approaches could transcend into multiple areas. For example, COVID and SARS-CoV-2 detection dogs, if the dog is trained to detect the disease state produced by the human in response to infection, that would fall into the medical detection dog category, however, if the dog is trained to detect the viral proteins produced during the course of infection, i.e., the etiological agent, then that would fall into the biological detection dog category (see Figure 1 for example).

Why the convergence of terms? Because the line between biological and medical is increasingly becoming blurred. COVID-19 brought this to the forefront as there were dogs trained to detect the COVID-19 disease state and dogs trained to detect the pathogen, SARS-CoV-2. Viruses are not living without a host and we are not training the dogs to detect the actual whole virus particles, or the culture media, but the viral proteins are produced by the host, with the odor resulting from both virus (pathogen) and human (host). When we consider if this falls into the biological or medical detection, it seems to fall squarely into biomedical detection. Perhaps until we know more as to what exactly the dog is detecting odor-wise, this broad category is appropriate. Or perhaps increased knowledge into what the dogs are detecting will only perplex us more as to how dogs are able to detect the signal from the noise in these incredibly complex backgrounds.

### What Are the Potential Applications of a Biomedical Detection Dog?

In most disease outbreak scenarios, biomedical detection dogs could serve a role in the detection of either the disease state or the pathogen directly. Early in the COVID-19 pandemic, before rapid diagnostics were widely available and once the general public started to become aware of the ability of canines to detect diseases and specifically COVID-19, prominent members of the canine detection community were fielding inquiries from all over the world on the potential applications and abilities of BMDDs. Table 3 lists many of the locations in which COVID-detection BMDDs either were (since 2020) or could be deployed in a disease outbreak scenario. The most obvious deployment scenarios were in transportation hubs such as airports and railway stations to quickly screen passengers during their travels in an attempt to stop the spread of the virus due to global travel. The majority of the scenarios involved active screening in which BMDDs would be actively searching the traveling public as they move from one location to another; however, other scenarios soon gained interest as institutions and businesses sought ways to not only re-open but to stay open during an ongoing pandemic. These latter scenarios called for BMDDs that monitor a relatively consistent resident population in a given area for changes in their infectious status. Since dogs are able to detect subtle changes in the volatilome (the odor actively being released), and often recognize signs of infection before and more accurately than traditional diagnostics, BMDDs offer a potential early warning system to alert us to the presence of an infected individual before they know they are ill, or before they demonstrate some of the more canonical signs of infection (e.g., fever, chills, aches, nausea, cough) (68, 80).

**TABLE 3:** Locations where biomedical detection dogs have or could be deployed during a disease outbreak.

Location(s)	Purpose/application
Schools (115)	One-time screening of visitors
Prisons	Periodic screening of traveling public
Work Sites/Buildings (116)	Confirmation of Negative COVID Tests for Entry
Ships (Naval, Cruises, Cargo) (117)	Surveillance screening of resident population (e.g., assisted living residents)
Assisted Living Facilities (116)	Daily screening of personnel (e.g., workers, teachers, students)
Farms*	Patient triage
Transportation Hubs (Airports, Railways) (78)	Sample screening
Border Crossings	
Hospitals (58, 118, 119)	
Mass Gatherings (e.g., graduation ceremonies, concerts, sporting events) (120)	

*Often populated by workers who do not have access to regular medical care or testing sites or fear repercussions associated with authority figures (e.g., deportation).

One of the most compelling use cases for BMDDs during a disease outbreak is in the medical care or hospital setting. BMDDs are capable of screening hundreds of people in a non-invasive manner, a sniff of the airspace around the person, in less than an hour. This capability can be used to triage long lines of people waiting to get tested or enter medical facilities. Instead of using an inefficient and hazardous first-come-first-served approach, the BMDDs can assist in identifying the people who are most likely positive for the disease, isolate them in a separate area, and expedite their tests. Selecting the presumptively positive individuals from the testing line, increases testing efficiency, removes the infection from the zone of susceptible people around them, and shortens that critical time to diagnosis (TTD) window which helps medical personnel take the proper disease precautions and administer the appropriate medical care, and allows faster allocation of limited medical resources (personnel and supplies).

Should a disease outbreak be so severe that PPE and test equipment were again to be in short supply or non-existent, BMDDs could also serve a role in helping triage the use of these items in the decision-making process before patient treatment. In this scenario, it is possible that it would be necessary to rely upon BMDDs to make the preliminary presumptive positive detection so that diagnostic tests are, in theory, only utilized on positive patients and thus the associated PPE, medical supplies, and testing equipment/kits would be prioritized and spared.

Disease outbreaks, epidemics, and pandemics can arise from different sources, either natural or “man-made.” Naturally occurring infectious diseases follow the typical chain of infection whereby disease transmission occurs when the pathogen leaves its reservoir and is transmitted to a susceptible host. For example, the disease malaria occurs when a Plasmodium (etiological agent) infected mosquito (reservoir) bites (mode of transmission) a human (susceptible host). Disease outbreaks could also arise due to human error or malintent such as an act of terrorism. Human error involving personnel working in high containment laboratories and poor biosecurity practices could lead to an accidental release of a pathogen into the environment. Faulty facility management, followed by a series of other major engineering control failures, could lead to negative pressure laboratories becoming positive pressure and resulting in a pathogen release. Intentional acts of bioterrorism could cause disease outbreaks as well. While the US Military, CDC and USDA (85) publish lists of biological warfare agents (BWAs) and Select Agents, with the growing popularity and ease of access to commercial-off-the-shelf synthetic biology laboratory kits, it is possible that one could weaponize a relatively benign microbe without much investment of time or money. Even without modifying a microorganism, acts of bioterrorism could be committed simply through strategic release of influenza or another common pathogen which would result in the destabilizing of the community.

Depending on the training aid and methodology utilized, it is possible to train BMDDs to search for infected patients, the etiological agent itself, the facility growing (biomanufacturing or culturing) the pathogen in the case of terrorism, or even odors associated with the production of the pathogen such as spent culture or growth media. It should be noted, however, that the process by which the breadth and specificity of these capabilities is accomplished is quite complex. Training a BMDD has many similarities as the training process for an explosives or narcotics detection dog; however, there are some unique considerations that must be made before, during, and after canine selection, training, and deployment. The topic of canine selection and performance considerations has been reviewed in depth by Lazarowski et al., MacLean et al., and others (86–92). Training a BMDD differs in the following ways:

• Typically requires numerous potentially infectious patient samples and/or a potentially hazardous training aid that requires specialized containment.
• Presumed that the odor of disease or a pathogen is not the salient odor in the scent picture, therefore training must be more nuanced to teach the dog how to discern the signal from the noise and normal from abnormal.
• Canine threshold must reach lower limits of detection as disease/pathogens produce less odor than most common canine training aids (e.g., narcotics and explosive training aids).
• PPE is often required during training aid handling and storage.
• PPE is often required during deployment.

Finally, the deployment concept of operations, the medical and legal ramifications of a BMDD alert, and how to handle discordant results between BMDD and diagnostics should be determined before utilizing a BMDD operationally.

### Deployment Scenarios

**FIGURE 2:**
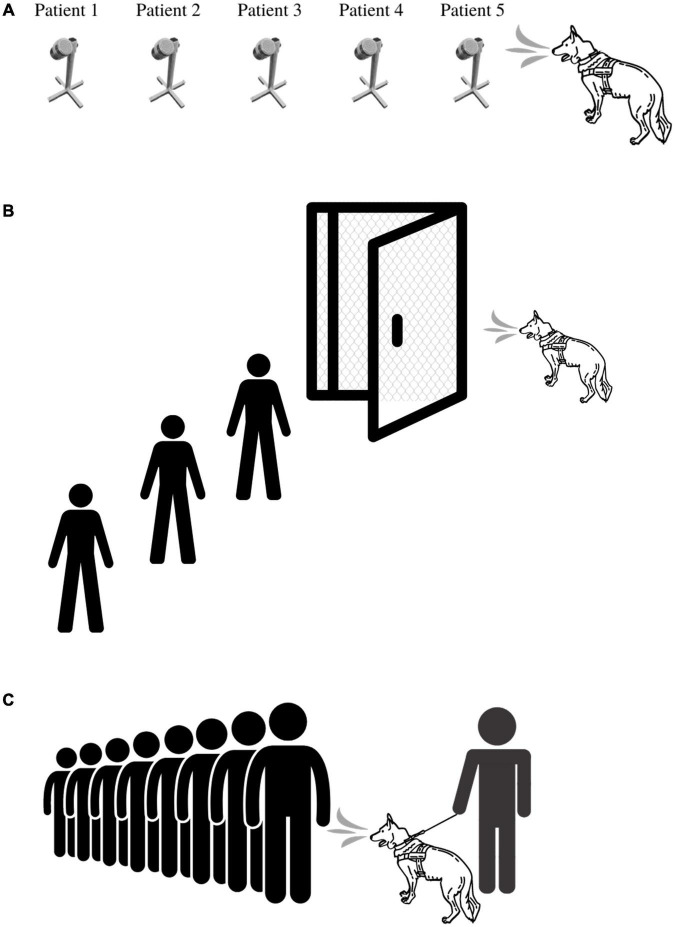
BMDD deployment scenarios utilized during COVID-19 pandemic. **(A)** Deployment Scenario 1 illustrates the most basic of deployment scenarios in which a BMDD screens people or environmental samples in an area separated from the disease outbreak. **(B)** Deployment Scenario 2 illustrates BMDD people screening in the disease outbreak area, yet physically separated from the population. Here, the BMDD is separated by a mesh screen or high efficiency particulate air filter if needed and can screen people through a checkpoint or individually through a lineup or room. **(C)** Deployment Scenario 3 illustrates the most complex deployment scenario in which a canine team screens people either en masse or in a lineup by being able to directly sniff each individual or group of people.

There are several ways in which BMDDs can be deployed during an outbreak. Figures 2A–C illustrates three of the primary ways in which BMDDs can screen humans for disease. The first scenario (Figure 2A) demonstrates a BMDD search of patient samples in a lineup. This set up has the least number of distractions for the BMDD as the search consists of discrete sampling points in the scent cans, a static odor presentation (i.e., the odor is not moving on a person in transit) which gives the BMDD adequate time to sample (sniff) the odor, and allows the sample collection team to reliably and reproducibly capture a sample from each patient/person. This scenario is the least hazardous of the deployment options as the BMDDs can be stationed in a separate room within the facility (e.g., airport, hospital, federal building) so there is no direct contact between the canine team and the public and/or the canine team and the patient samples. This scenario also eliminates potential allergic reactions to canines and interactions with people who fear canines.

The second scenario (Figure 2B) illustrates live human screening in a controlled manner in which people are individually searched by a BMDD behind a mesh screen/barrier. The humans individually enter a small room that is divided in half by a mesh screen barrier, the human is on one side and the BMDD is on the other side. Air flow would be established to flow from the human side to the canine side. The human is sampled or sniffed through the barrier and then leaves the room. This set up allows for physical separation between the patient and the BMDD while providing the entire human as an odor source for the BMDD. The scenario also maintains more control of the operational environment by limiting distractions and controlling airflow.

The third scenario (Figure 2C) is the most difficult deployment scenario of them all as limited control of the operational environment exists and therefore BMDDs do not obtain the same sample from each person screened. Indeed, utilizing the first BMDD scenario, three COVID-19 detection studies had pooled sensitivities and specificities of 0.88 (95% CI, 0.84-0.91; I2, 85.3%) and 0.99 (95% CI, 0.99-0.99; I2, 97.4%), respectively (82); however, when one research group attempted to utilize the third deployment scenario, the positive predictive value plummeted to 28.2% (59). Jones et al. discuss the intricacies of screening travelers, specifically for COVID-19, in their perspective paper, “Could bio-detection dogs be used to limit the spread of COVID-19 by travelers?” (93) calling for additional research while also discussing plans for the next phase of their study in which sensitivity and specificity of BMDDs will be assessed at COVID-19 test centers where they can sniff individuals waiting to donate swab samples for formal diagnosis. This study will provide much needed data to the body of literature for this difficult deployment scenario.

In general, one should first consider the deployment zone and whether the BMDDs will have direct person/patient contact, be segregated by a physical barrier that still allows for scent detection or be kept in a room used exclusively for scent detection lineups. Choosing the deployment method should further take into account a thorough risk analysis (to include legal and medical ramifications), the culture of the people being screened, how to sample people who are allergic or fear canines, and public perception and acceptance of canines (52). Two of these BMDD deployment methods were utilized during the COVID-19 outbreak. Private companies within the United States deployed canines at sporting events (Figure 2C) and the United Arab Emirates (UAE) utilized canines in airports keeping the canines in a dedicated sample screening room away from travelers (Figure 2A).

Once a patient sample or person is alerted on by the BMDD, if one is available, a diagnostic test should be performed to confirm the BMDD’s detection response; however, it should be noted that the BMDD may have detected an earlier stage or asymptomatic presentation of infection that the diagnostic test will not be sensitive enough to detect.

### Safety Considerations

Several safety considerations should be made before, during, and after the utilization of BMDDs. Specifically, a safety hazard analysis should be conducted to weigh hazard probability vs. hazard severity and create a decision matrix in which the overall risk of the operation (i.e., BMDD deployment) can be characterized. In this matrix, hazard probabilities range from unlikely, to seldom, occasional, likely, and frequent, while hazard severities range from negligible, to moderate, critical, and catastrophic (94). Pre-deployment medical screening of canine and handler, periodic testing (antibody and/or antigen) of canine team, and a system in place for daily monitoring of clinical signs, should all be established and maintained. While the human or canine may not be the ideal host initially, during an outbreak as pathogens mutate, pathogen host ranges may expand, hence the importance of ongoing disease screening of the canine team (dog + handler).

Part of risk management is developing and implementing controls. Controls should be evaluated during the decision-making process and implemented in several areas along the way toward a deployed detection capability. Depending on the type of disease outbreak, one may not have the option of deciding whether or not they are going the route of developing a BMDD as the nature of the etiological agent may dictate this path. For example, if faced with a highly pathogenic avian influenza outbreak that was capable of infecting birds, pigs, humans, and dogs, this pathogen would most likely be classified as a biosafety level four (BSL-4) organism, the handling of which would be limited to just a few dozen laboratories between North America and Europe. It is highly unlikely in this scenario that the medical community would have the resources to be supporting the canine community with patient or virus or virus-derived samples, and that the public health community would allow potentially infectious material to leave high containment laboratories. One factor working in favor of BMDD training aid creation, however, was published by Jendrny et al. when they observed that chemically inactivated (beta-propiolactone) SARS-CoV-2 saliva, urine, and sweat clinical samples could be used BMDD training and subsequently the dogs could generalize their detection capabilities to non-inactivated clinical samples and even to other body fluids (66).

### Training Aids

**FIGURE 3:**
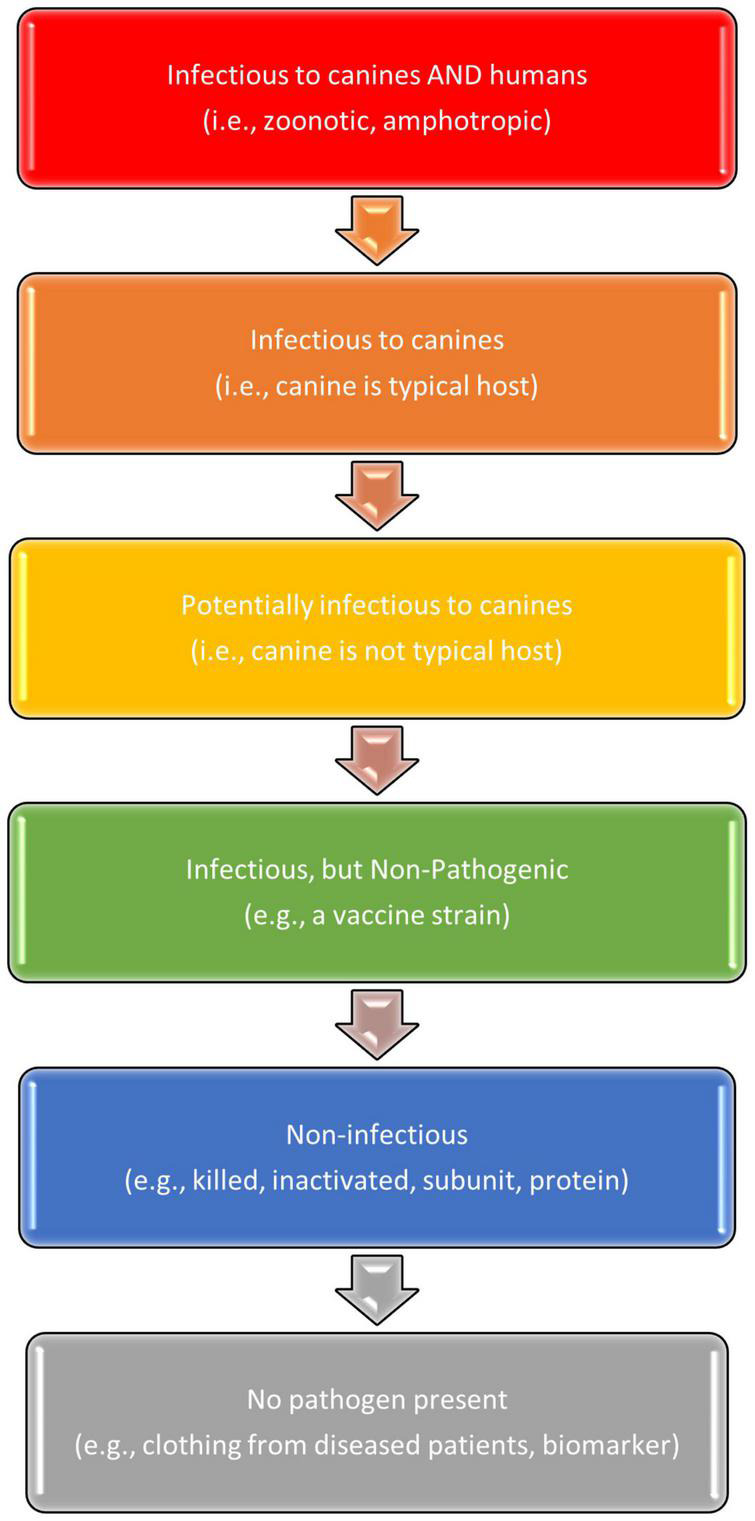
Selection of canine training aids for biomedical detection dogs.

Zoonotic outbreaks, however, should not *necessarily* preclude the development of BMDD training aids and eventual BMDD deployment. This is due to the fact that risk mitigation steps such as the aforementioned deployment scenarios, containment of the aid in the SciK9 (Lorton, VA) training aid delivery device (TADD), and/or odor ad/absorption-based training aid technologies, can be used in conjunction to mitigate risk and create a safe-to-handle training aid regardless of the hazard-class of the etiological agent. Figure 3 illustrates the hierarchy of choices from most (top) to least (bottom) hazardous for the selection and development of canine training aids for BMDDs. Ideally, the outbreak pathogen would be categorized in the lower half of this diagram. It is important to remember that the premise of BMDD technology relies upon canine detection of odor, i.e., the VOCs emanating from the source and not physical contact with the actual pathogen or samples from a diseased human. Thus, by instituting proper safety and risk mitigation strategies, BMDD deployment may still be an option scientifically and operationally regardless of infectiousness of the disease outbreak pathogen.

In the disease outbreak scenario, BMDD training aids are typically divided into two categories depending on if the goal is pathogen detection as in the case of a biological detection dog (BDD), or disease detection as in the case of a medical detection dog (MDD) (Figures 2A–C). Training aids developed for the former can potentially consist of purified pathogen, pathogen culture, cell culture supernatant, spent cell culture media, inactivated pathogen (*via* heat, steam autoclaving, or chemical inactivation methods), modified pathogen (*via* utilization of existing vaccine strains, genetic engineering, or attenuation through passaging), and biomolecular components or metabolic products of the pathogen that produce a representative signature pathogen-specific odor (proteins, oligosaccharides, metabolites, envelope or membrane-associated lipids).

Training aids developed for MDDs can consist of direct capture of bodily fluids (e.g., urine, blood plasma, blood serum, sputum, nasal swabs, saliva, feces) or human scent (breath, sweat, skin/body odor) captured onto a substrate (clothing, gauze pad, cotton ball, worn surgical mask) from infected and uninfected patients (65, 77). The canine training aid(s) selected for a BMDD is of utmost importance as this decision will affect the canine’s ability to detect the target odor (either disease or pathogen) and may influence the canine’s ability to generalize to other target odors, e.g., novel patient samples, and discriminate from other similar pathogens, e.g., non-pathogenic strains of a virus or bacteria, both highly desirable BMDD skills. BMDD training aid selection, development, shelf-life, service-life, comparative analysis of efficacy and efficiency, and associated training methodologies and standards, and are all areas in dire need of research as each scientific group around the world took disparate paths in their approach to developing a COVID-19 detection dog capability.

### Containment

**FIGURE 4:**
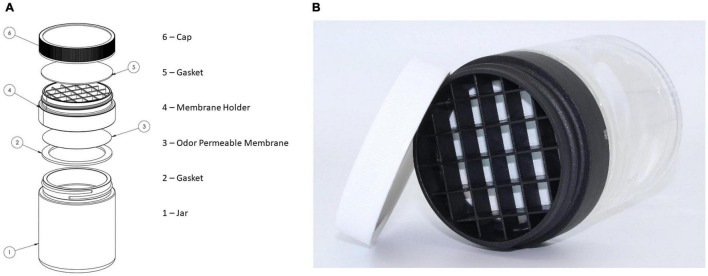
Training aid delivery device (TADD) breakout diagram **(A)** and photograph **(B)**.

The training aid delivery device (TADD) by SciK9 (Figures 4A,B) is a primary containment system for canine training aids that physically secures the training aid substance inside while allowing the odor out through a gas-permeable membrane (95). The TADD’s membrane has hydrophobic and oleophobic qualities that allow for liquid and solid training aids typically required of BMDDs, such as blood, urine, or feces. Meanwhile, the TADD’s membrane holder protects the membrane from physical penetration by the dog or handler as well as protecting the training aid from the operational environment of canine training, thus protecting precious clinical samples such as biopsy tissue or oropharyngeal swabs. The TADD facilitates the training of BMDDs on potentially hazardous materials such as their training aids in a safe manner while also protecting the handler, trainer, and the BMDD during the training process. The TADD was utilized during the training of COVID-19 BMDDs to protect the dogs from potential exposure to SARS-CoV-2 and any other pathogens that may be present in the patient samples (76). Another group tested the TADD for its ability to contain the SARS-CoV-2 virus by swabbing the outside of TADD-membranes after each day of canine testing and performing RT-PCR-assays to exclude virus escape (66). The TADDs demonstrated were swabbed and tested sixty-eight times, which resulted in 68/68 negative PCR reactions, thus demonstrating 100% containment of the hazardous biological material (66), while also enabling the BMDDs to successfully train on the odor of COVID-19 and detected non-inactivated saliva samples with a diagnostic sensitivity of 84% and specificity of 95%. Furthermore, in subsequent experiments the BMDDs were able to detect three non-inactivated body fluids with similar accuracy, achieving a diagnostic sensitivity and specificity of 95 and 98% for urine, 91 and 94% for sweat, 82, and 96% for saliva, respectively (66).

### Odor-Ad/Absorption Based Training Aids

Odor ad/absorption-based training aids present a safe and reproducible way to capture and release a variety of odors from potentially hazardous material. From the traditional odor soaks utilizing natural fibers like cotton balls or towels to the latest polymer-based absorption training aids, these substrates allow training aids to be transported, stored, and handled without special requirements or permits typically mandated when dealing with hazardous material, and because they only contain the odor of the pathogen or disease state and are not infectious in nature, they present a safe alternative to training dogs to detect potentially deadly pathogens.

**FIGURE 5:**
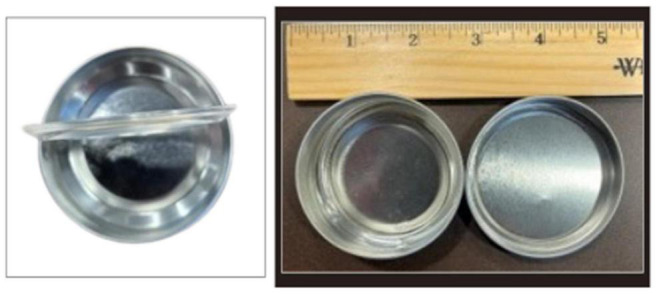
PDMS based Odor-Ab/adsorption canine training aid depicted in a small metal sniffer tin.

National Institute of Science and Technology (NIST) pioneered the polymer-based adsorption canine training aid work in their development of a polydimeythlsiloxane (**PDMS)-based training aid** for explosives (96, 97). The PDMS-base training aids (Figure 5) are non-toxic, non-infectious, and can be impregnated with nearly any odor, making the potential for this training aid technology nearly limitless for BMDDs. Based on the published research demonstrating the steady odor release rates of explosive material over time and recent method development publication on the rate of odor capture for less volatile targets (98), it stands to reason that this technology should be investigated for PDMS applicability in the creation of biological training aids.

**FIGURE 6:**
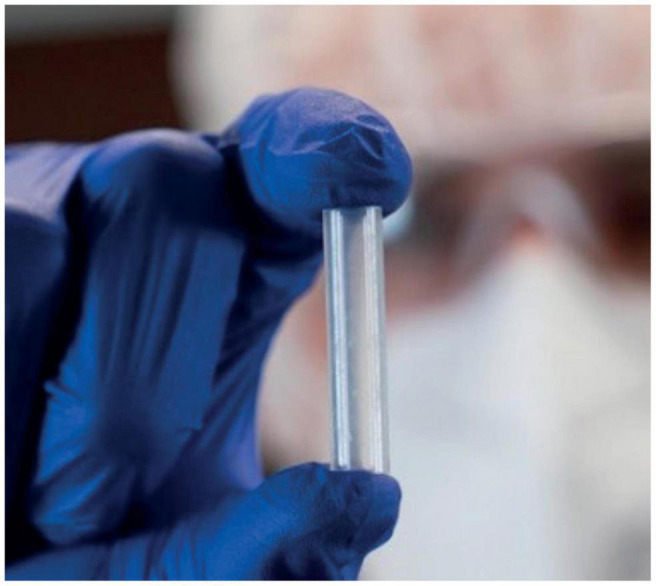
Photograph of getxent odor adsorption tube.

**Getxent tubes** (Figure 6) represent another odor-absorption technology for the creation of canine training aids. The small tubes with an outer diameter of 8.0 mm, inner diameter of 5.4 mm, and length of 35 mm, are made of a proprietary blend of copolymers (certifiable biocompatible USP class VI), containing both polar and non-polar blocks allowing the absorption, storage, and release of odorous molecules (VOC) (99). They are supplied odorless and can be impregnated with target odor by co-incubating the Getxent tube within the headspace of the substance of interest. Getxent tubes were utilized extensively during the COVID-19 pandemic for quickly sampling the axillae of COVID patients during the research phase of canine training and while screening the traveling public during the BMDD deployment phase (74).

**FIGURE 7:**
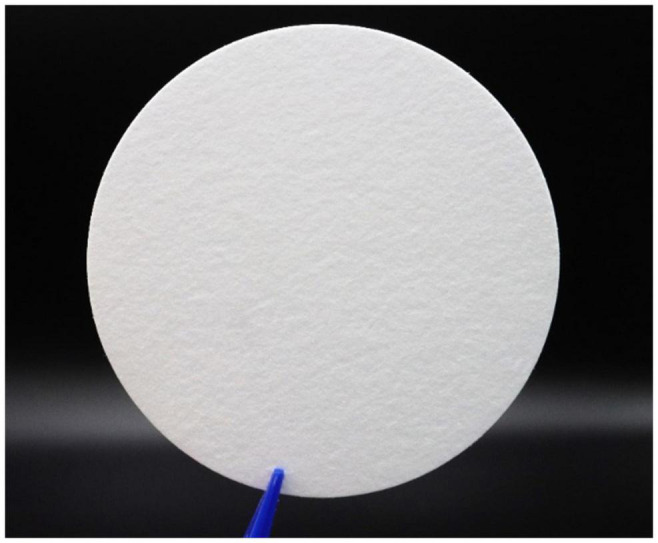
Cellulose microfiber based filter paper for odor soaks.

**Traditional odor-soaks** may also be considered and have been demonstrated as efficacious in explosives, narcotics, and many other areas of canine detection. This method involves impregnating target odors *via* co-incubation in the headspace of the substance of interest with laboratory-grade glass, cellulose, or paper microfiber papers (Figure 7) (approximately 25–40 mm diameter pieces recommended) (100).

For all of the odor ad/absorption-based training aids, if the training aid is infectious or hazardous in nature, it is recommended that an appropriately sized filter barrier be placed between the training aid (e.g., pathogen (virus, bacteria), diseased tissue, field sample) and the odor-soak substrate to prevent accidental contamination of the substrate with aerosol or particulate from the potentially hazardous sample.

### Safety Measures and Decontamination

**FIGURE 8:**
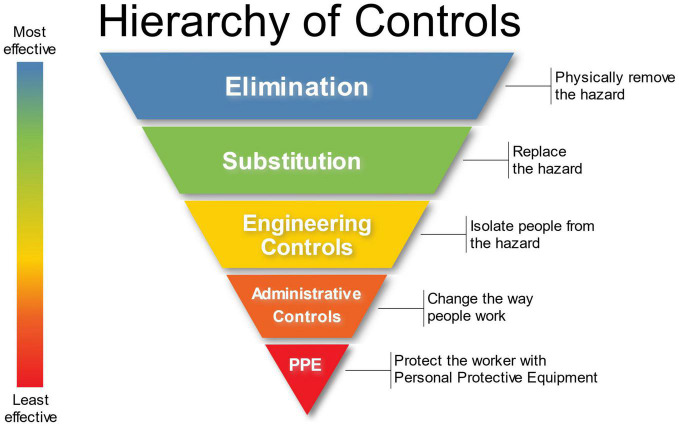
Hierarchy of Controls in the Environmental Health and Safety Paradigm. Attribution: Original version: NIOSH Vector version: Michael Pittman, NIOSH’s “Hierarchy of Controls infographic” as SVG, CC0 1.0.

Other protective measures that should be considered based upon a thorough risk assessment is the utilization of canine and human PPE. The same hierarchy of safety and health controls for healthcare personnel (Figure 8) should be applied to biomedical detection dog teams. BMDD handlers should receive proper education and training with respect to the pathogen or disease they are being asked to detect as a canine team. At a minimum, trainers and handlers should be able to recognize signs of disease, understand modes of transmission, any PPE required, and how to respond to a potential exposure. This education and training will help keep canine teams safe during training and operations. Industrial hygienists, professionals specializing in environmental and occupational health and safety, should be consulted to assess the engineering controls that can be instituted and should be emplaced. Industrial hygienists can also help develop workplace practice controls such as when and how to wash uniforms/clothing or the prohibited actions such as smoking, eating, and drinking in the work environment.

The biological risk assessment will help determine the proper PPE to utilize in addition to the appropriate work practices and containment requirements. This part of the risk assessment considers the properties of the biohazardous material such as pathogenicity, infectious dose, host range, agent stability and viability in the environment, availability of preventative therapies (e.g., vaccines), availability of post-exposure prophylaxis (e.g., immunoglobulin therapy), potential outcomes of exposure, and routes of exposure (inhalation, ingestion, dermal, or injection).

It should be noted that before BMDD utilization, both the canine and human should have individual risk assessments, followed by a joint canine team assessment. This approach will ensure that the canine-specific risks are being evaluated and avoid an incomplete or anthropocentric risk assessment. Canines face different risks than humans during operations. For example, canines are lower to the ground and will encounter different exposure hazards, as they are unable to utilize face-filtering respirators and masks whilst performing their scent detection duties, and rely entirely on the handler to keep them safe, having no concept of the risks involved.

Decontamination of the canine team and any associated equipment should be considered and planned for in advance of deployment. The effects of serial decontamination of canines are unknown at this time, so great care should be taken to avoid compromising the skin barrier by repeatedly washing or wiping canines. Only veterinary approved solutions should be applied to canines in an effort to avoid irritating or damaging canine skin and mucosa and avoid any potential toxicity issues. Dr. Erin Perry at Southern Illinois University published guidance on how to effectively decontaminate canines using two different methods, one in which water would be freely available and the other wherein water would not available and thus wipes are used to decontaminate the canine (101). The wipe-down procedure utilizing dilute povidone-iodine scrub wipes was later validated as the superior method for removing generic aerosolized particulate from canine coats when compared to dilute chlorhexidine-gluconate scrub wipes or water (102). While these studies provide information on the bulk removal of aerosolized particulate, it is still unknown if the chemicals in the decontaminant solutions are able to neutralize chemical or biological threats on canine fur as these decontamination solutions were developed and optimized on and for bare skin with the human end-user in mind.

Currently, Chemical Biological Radiological Nuclear (CBRN) decontamination recommendations for military working dogs are vague, lack a standardized protocol, and fielded kit, and are based upon the obsolete Army Field Manual 4–02.18 from 2004 (103). The U.S. Army’s Public Health Center is currently conducting studies to address this knowledge gap. In conjunction with the US Army’s Combat Capabilities Development Command—Chemical Biological Center (DEVCOM CBC), toxicologists, microbiologists, canine subject matter experts, and decontamination scientists are working together to validate the efficacy of two decontamination methods, the established method for chemical threats from Army Techniques Publication (ATP) No. 4–02.85 requiring access to water and a field-expedient low-water approach utilizing wipes and microfiber cloths (104). It is important to note that the military research and guidance will likely be directed toward acute exposure scenarios and decontamination and medical management of military working dog casualties. This is in contrast to the chronic exposure scenarios likely to be encountered by search and rescue (SAR) canines that Dr. Perry addresses in her decontamination guidance.

Due to the often disparate canine decontamination recommendations, it is therefore advisable to create a strategy for how personnel will handle potential exposure scenarios to both themselves and their canine partners and what measures should be taken on a daily basis to ensure that the canine does not become a fomite or disease transmission source.

The variance in how canine training is approached from trainer to trainer, amongst academic institutions, between countries, and from one detection discipline to another is staggering. One of the first research needs for BMDDs is standardization. If BMDD-based detection is going to have a role in the next disease outbreak scenario or a role in future medical diagnostics, there need to be established standards. Edwards et al., in their 2017 publication entitled “Animal olfactory detection of human diseases: Guidelines and systematic review,” outline the ideal training, testing and operational conditions for working with BMDDs and the associated samples (19). The authors provide recommendations regarding the type and depth of information that should be included when describing BMDD research so that these studies can help the scientific community compare the utility of detection methods for specific diseases or pathogens (19). Then, standards need to be brought to the human medical, diagnostic, public health, and regulatory communities to validate that the consensus publication standards will meet the evidentiary needs of a new detection/diagnostic-based technology.

From a training perspective, canine trainers should be prepared to deal with frequent and periodic quality checks and assurances on their BMDD. This is necessary because canine generalization in the operational environment works both for and against the detection capability. Generalization capability can potentially allow BMDDs to detect different disease clinical signs, different strains of a pathogen, asymptomatic or pre-symptomatic presentations, and diseases regardless of the patient age, sex, race. Yet generalization could also go beyond the detection capability we desire and potentially lead to BMDDs detecting related but non-pathogenic microbes, unrelated microbes, non-infectious disease presentations, non-specific fevers, etc. Beyond certification, BMDDs will need to be recalibrated on their training aids often in order to ensure that they are still proficient on target odor. Additionally, BMDDs should also be certified using distracting odors from other diseases/pathogens to ensure that the dogs are still adequately discriminating target from non-target odor. Finally, during deployment, when the infectious status of a person is unknown, the reinforcement schedule of the BMDD must be carefully considered so as to not incorrectly reward the dog. There are several ways to address this, however, they are beyond the scope of this article.

Cooperation is also needed on the macro-level like the application of the One Health ideology wherein the veterinary and medical communities from the local to regional, national, and global levels communicate and collaborate to support for the research, development, test, and evaluation (RDT&E) needed to address current gaps in BMDD research, overcome BMDD deployment concerns, pair BMDDs with electronic sensors, and strategize how to scale-up operations for the next pandemic.

### Research Needs

Immediate research needs to be conducted on the shelf- and service-life of biologically derived and patient-derived canine training aids, methods of sample containment, storage, and preservation, and best practices for characterizing BMDD training aid samples. Further research is also needed to determine what the dogs are actually detecting. A pared down, process-of-elimination approach combining systematic headspace analysis combined with canine olfactometry such as the one present at Dr. Nathan Hall’s Texas Tech University Laboratory (105) could lead to biomarker discovery, a VOC-responsive colorimetric sensory array (106), or to the understanding that BMDDs are capable of far more than we have realized and a significant investment in the understanding of odor has enormous potential not only for once in a lifetime pandemics, but for breath-based diagnostics in the primary case setting, detection of invasive pests in big agriculture, assessment of the human volatilome for stress, fatigue, anxiety, and many other use cases.

Another research need is a holistic view and comparison of the medical detection dog and biodetection dog approaches to a disease outbreak. For example, during the COVID-19 pandemic, nearly all BMDD research groups around the world took the MDD approach, training dogs to detect the disease state of COVID-19 using patient clinical samples as canine training aids (52). However, a private business within the United States pursued the BDD approach, training dogs to detect the SARS-CoV-2 virus using viral proteins as canine training aids (107, 108). The BDD strategy rationale provided by the private business was based upon a discovery made with agriculture detection dogs studying one of the most severe pandemics in modern times, huanglongbing (HLB) disease of citrus, caused by the bacterium *Candidatus Liberibacter asiaticus* (*C*Las). Gottwald et al. observed that canines were detecting *C*Las bacteria directly rather than only host volatiles produced in response to infection and demonstrated this when the detection dogs identified *C*Las-infected tobacco, periwinkle, psyllid insect vectors, and bacterial co-cultures (109). The BDD approach utilized by Gottwald et al. and subsequent deployment of COVID-19 BDDs by private businesses within the US warrants additional investigation especially as this method has some advantages over the MDD approach.

The MDD approach to detection during an outbreak requires intensive recruitment of suitable subjects of both positive and negative disease status, and samples that represent the infected population in frequency, age, sex, ethnicity, and overall health status (access to healthcare, comorbidities, etc.) (19, 82). The MDD approach also requires contact with and handling of infectious patient samples, rendering the samples safe either by physical, chemical, and/or containment means, training dogs on up to hundreds of these disease positive and negative samples, special storage conditions for the samples or constant access to single-use samples, and great coordination amongst personnel that are typically not co-located (i.e., hospital staff and canine trainers) (81, 82, 110). This approach requires follow-up to ensure that patient infection status has not changed, e.g., a previously negative patient whose sample was collected for canine training became symptomatic and then tested positive 48 h later. This scenario does occur and needs to be controlled as patient-derived canine training aid samples are heavily relied upon to be negative or positive. The MDD approach also requires that canine training aids have patient history and demographic data to accompany each sample so that a representative cross-section of the population can be surveyed and presented to the dogs. These canine training aid samples should be assigned a unique number and firewalled from the patient data, i.e., deidentified, to preserve the privacy of research participants. Sample and patient information should include the data outlined in Table 4. The information provided in Table 4 could easily be adapted for MDD studies involving agricultural detection dogs and plant diseases in which similar information about the age, growth conditions, health, and disease status of the plant would be important to know.

**TABLE 4:** Minimum sample and patient information recommended for biomedical detection dog studies.

Sample	Examples of additional information
Unique identifier	Ties sample to patient data in a way that no patient or sample information can be gleaned from the identifier.
Type	Sputum, urine, blood, culture, insect casings, viral proteins
Suspension	Buffer, glycerol, media, formaldehyde, formalin, none
Sample capture Substrate/matrix	Swab, cotton pad, odor-absorption, none
Duration of sample collection	Length of time ventilating a surgical mask, duration of an odor-absorption tube in a patient’s axilla (armpit), or co-incubation time of a filter paper to create an odor soak with the target substance
Infectious status	Live, inactivated/killed (state inactivation method), attenuated, non-hazardous
Sample containment	Serum separator tube, metal sniffer tin, urine collection cup, TADD, glass jar
Odor contributing sources	Gloves, masks, permanent marker
Time, date of sample collection	14:00, 2020-12-30
Time, date of sample receipt	
Time, date of sample Analysis	
Time, date of sample Storage	
Time, Date of Sample K9 Testing	
Collection Setting	Home, Diagnostic Lab, Research Lab, Hospital, Doctor’s Office
Collector	Person who collected the sample, e.g., Patient or medical professional’s name
Transport method and conditions	Shipped overnight cold storage or ground transport at ambient conditions?
Storage conditions	Location, temperature, humidity, any other unique conditions (e.g., vacuum storage, with desiccant, segregated positive from negatives, etc.)
**Patient demographic data**	**Patient history data**
Date of Birth	Current disease status
Age Range	Confirmed test result(s) for disease of interest
Ethnicity	Type of test(s) performed
Race	Date of testing
Sex	Date of results
City, State	Date of results notification
Type of Housing	Current symptoms
(e.g., detached home, apartment, communal living, etc.)	
Cohabitation with animals	Chronic health conditions
Cohabitation with human and their disease statuses	Positive for disease of interest in the past?
	Vaccination status for disease of interest
	List of current medication
	Pregnancy status

The BDD approach, such as in the case of a SARS-CoV-2 detection dog (assuming the training aid is viral protein and is efficacious resulting in a detection capability on COVID-19 positive patients) could have several advantages. (1) Unlike most detection or diagnostic laboratory-based equipment, canines have the unique ability to generalize and expand their “library” of target odors. If there is enough similarity between the odor (not the nucleic acid or amino acid sequence) of the current training aid and the odor of the circulating strains, that would be sufficient for the canines to alert. (2) The VOCs produced by the human immune response to an infection will eventually become part of the scent picture to the operational biodetection dogs, thus, in addition to the training aid, they will also have odors “in theater” that could allow for a persistent and enduring capability. (3) Canines have an incredible ability to find the novel odor in a familiar environment, called neophilia, and are known to find and detect anomalies due to this phenomenon. One theory is that since the viral proteins are novel to the canines, they are easily detectable compared to the environment and background odor. This novelty/anomaly could supersede and overcome the protein differences caused by viral mutation. The proteins would continue to be classified by the canines as “within the same odor family” as their training aid, such that the canines can generalize and alert to the proteins produced by the virus variants. (4) Since the training aid is laboratory-made, cultivated in cell culture and purified virus protein, it can be re-formulated, modified, and multiplexed to include additional strains, variants, and proteins.

Following the SARS-CoV-2 biodetection dog example, there are also limitations that should be noted. Assuming that the training aid is composed of antigenic spike proteins, these protein sequences are constantly mutating as the RNA virus evolves. This could then require continuous reformulation of the training aid to ensure the composition/odor is representative of the circulating strain(s) of the virus. There are potential limitations should the disease outbreak be caused by a prion, whereby the infectious material is itself a protein and perhaps any attempts at modifying the protein to render it non-infectious alters or obliterates the odor profile, thus rendering any training aid ineffective. Finally, the biggest drawback of this approach currently is that it was the unconventional path, pursued by a private company in one country, and not third-party evaluated, while comparatively the MDD approach was pursued globally and successfully demonstrated by well-established research groups and published in peer-reviewed journals, therefore the biodetection dog approach, at least for its utility in a human disease outbreak scenario, is higher risk and unknown at this time. Due to the potential advantages, however, the biodetection dog approach should be considered and compared to the MDD approach so that as many detection tools as possible exist for the next disease outbreak.

One area for improvement is the development of consistent and communicated training and testing protocols, making it possible for external parties to understand how successful dogs are at detecting an odor in both laboratory and real-world situations. Basic sensitivity and specificity reports do not completely inform readers about the conditions at time of testing (111). Different distractors may cause increased false alerts, testing scenarios may be less controlled than training leading to reduced true positives, and lack of blinding for trainers or testers may cause artificially high detection rates. We recommend stating if handlers and/or test administrators/observers are blind to target locations, communicating the type and number of non-target odors, and providing tables of test results in addition to overall sensitivity and specificity numbers. The criticality of not only publishing detailed protocol information, but also noting and tracking this information for each training and testing sample was highlighted by Guest et al. in their publication “Subtle Aspects of the Processing of Samples Can Greatly Affect Dogs’ Learning” (112). To summarize, six dogs were trained to discriminate between hospital-sourced target urine and externally sourced control urine believed to be processed and stored the same way. During initial testing, dogs displayed good accuracy with a mean sensitivity of 93.5% (92.2–94.5) and specificity of 87.9% (78.2–91.9). However, upon further testing, when samples included hospital-sourced controls, the dogs performance greatly decreased in specificity 67.3% (43.2–83.3). Upon further investigation, it was found that the two sets of samples varied in one critical aspect—sample processing. The hospital-processed samples were tested by dipping a urinalysis stick into the sample, while the externally sourced samples were tested by pouring a small amount of urine over a urinalysis stick. Dogs had learnt to distinguish the target samples aided by the odor of this stick. This highlights the importance of considering every aspect of sample processing, but also pertains to sample collection, storage, handling, shelf-life, and presentation.

## Conclusion

BMDDs offer a mobile, autonomous, non-invasive screening approach that provide real-time detection results in an efficient, reagent-free, and cost-effective manner. Furthermore, BMDDs can rapidly screening large numbers of people, samples, or areas, with a high degree of accuracy. But the one thing that BMDDs do that none of the other traditional screening or diagnostic tools can do is locate the target odor, find the infected person, source the unique signature volatilome, or alert to the most minute signal of a biological odor amongst the vast array of biological noise present in the operational environment. This “find” function combined with the ability of BMDDs to quickly clear the non-diseased patients/area, makes the potential for BMDDs unmatched in a disease outbreak scenario.

The limitations to BMDDs are broken down into those that are inherent in any scent detection dog discipline and those specific to BMDDs in a disease outbreak scenario. BMDDs themselves are living beings with the need for defined duty cycles to account for rest, sleep, eating, play, and all of the other needs of a canine. While rare, BMDDs have “off” days and thus it is advisable to have more than one BMDD in critical screening situations. And for now, we consider BMDDs a “closed system” in that they do not provide identifying information as to what they are detecting and instead simply provide a yes/no alert. Before a BMDD is ready for deployment there has already been considerable investment into the breeding, genetics, working dog criteria selection process, early neurological stimulation, early socialization training, and that is all in addition to standard rearing, veterinary care, and odor recognition training. Once a BMDD is trained and ready for deployment, in any scenario where they would need to be on-leash, such as Figure 2C’s deployment scenario 3, the BMDD requires a skilled handler to work together as a team during people or area searches. There is a plethora of other potential limitations, but most can be overcome with additional training and therefore are not considered inherent to BMDDs.

The limitations specific to BMDDs in a disease outbreak scenario are numerous in that many boxes must be checked before it can be done responsibly. Getting to the point of BMDD deployment takes enormous amounts of intergovernmental cooperation, effort, and coordination from access to patient samples to the navigating the legal aspects of people searching. Taking the MDD approach requires enormous effort dedicated to patient recruitment, testing, follow-up, sample remediation, characterization, storage, and containment, and all together, these endeavors require massive amounts of documentation, animal use protocols, institutional review board approvals, and coordination amongst medical, veterinary, and canine training personnel. Finally, without certification standard(s) specific to BMDDs in place, it will be difficult to install BMDDs in a way that instills public trust in the true capability of these incredible animals.

The potential of detection dogs during a disease outbreak is that they offer a promising strategy to addressing a gap in detection; however, to reach their full potential significant research investment in olfactory sciences will be required and the dividends will be substantial as the scientific outcomes will impact medical diagnostics, electronic breath-based sensors in public health, and stand-off detection technologies for hazardous materials.

## Author Contributions

All authors contributed to the writing of this manuscript and read and approved the final manuscript.

## Conflict of Interest

MM was the inventor of the training aid delivery device. MM and JG had minority partnerships in SciK9 LLC. MM and CS were employed by Excet, Inc. JG was the owner of Intrinsic24, LLC. PN was the owner of Tactical Directional Canine Systems LLC. The remaining authors declare that the research was conducted in the absence of any commercial or financial relationships that could be construed as a potential conflict of interest.

## Publisher’s Note

All claims expressed in this article are solely those of the authors and do not necessarily represent those of their affiliated organizations, or those of the publisher, the editors and the reviewers. Any product that may be evaluated in this article, or claim that may be made by its manufacturer, is not guaranteed or endorsed by the publisher.
